# Identification of Novel Genetic Loci Related to 100-Seed Weight in the Korean Soybean Core Collection Using a Genome-Wide Association Study

**DOI:** 10.3390/ijms262411921

**Published:** 2025-12-10

**Authors:** Ju Yeon Moon, Sangjun Park, Soo-Kwon Park, Jung-Kyung Moon, Jin A. Kim, Mi-Suk Seo

**Affiliations:** 1 Digital Breeding Convergence Division, Department of Agricultural Biotechnology, National Institute of Agricultural Science, Rural Development Administration, Jeonju 54874, Republic of Korea; jymoon7191@korea.kr; 2 Upland Crop Breeding Division, Department of Upland Crop Sciences, National Institute of Crop Sciences, Rural Development Administration, Miryang 50424, Republic of Korea; sksmszhzh@naver.com (S.P.); sookwonpark@korea.kr (S.-K.P.); moonkj2@gmail.com (J.-K.M.); 3 Plant Biomaterials and Biotechnology Division, Department of Agricultural Biology, National Institute of Agricultural Science, Rural Development Administration, Jeonju 54874, Republic of Korea

**Keywords:** soybean, 100-seed weight, high-density SNP, GWAS, haplotype

## Abstract

Soybean [*Glycine max* (L.) Merr.] is a commercially important oil and protein-producing crop. This study explored genetic variation in seed traits to improve soybean productivity. Phenotypic data, including seed size, length, width, thickness, and weight, were analyzed across 344 Korean soybean accessions, identifying 100-seed weight (100SW) as one of the important yield components in soybean. Using 4,472,823 high-density single-nucleotide polymorphism (SNP) markers, a genome-wide association study detected 10 novel loci associated with 100SW on chromosomes 3, 4, and 19. Haplotype analysis revealed that the accessions with alternative alleles at nine candidate loci displayed significant variation in 100SW, either increasing or decreasing weight. Allele stacking analysis further highlighted that favorable variants, particularly on chromosomes 3 and 19, had additive genetic effects on 100SW. Functional annotations suggest these genes influence seed weight through gibberellin synthesis and developmental pathways. By leveraging high-density genomic data, this study overcomes the limitations of previous studies relying on low-density markers, offering a foundation for more efficient soybean breeding strategies. These findings offer valuable insight into marker-assisted selection, providing a foundation to enhance soybean yield and adaptability under diverse environmental conditions and addressing the growing global demand for sustainable food production.

## 1. Introduction

Soybean [*Glycine max* (L.) Merr.] is a major crop cultivated for its edible oil and protein. Improving grain yield remains a top priority in soybean breeding practices worldwide [1]. Understanding the genetic architecture of quantitative traits in crops is a key focus of modern agricultural research, as it plays a crucial role in enhancing yield, resilience, and nutritional value. Among these traits, seed weight stands out as one of the most important agronomic determinants of soybean yield [2,3]. Seed weight, usually expressed as 100-seed weight (100SW), is a complex quantitative trait controlled by multiple genes/loci and constrained by environmental factors [4,5,6,7,8].

To date, numerous quantitative trait nucleotides (QTNs) and quantitative trait loci (QTLs) associated with soybean seed weight have been identified, highlighting key loci on multiple chromosomes and revealing several candidate genes that play significant roles in regulating seed weight across various environments [9,10,11]. However, many 100SW loci have been mapped to large genomic regions, often due to low-density markers, small mapping populations, or limited recombination, which complicates the identification of candidate genes. Only a few genes associated with seed weight and size have been validated, and most of the genes underpinning QTLs of soybean 100SW remain unidentified. Huang et al. (2021) identified four candidate genes, including *Glyma.04g047800*, *Glyma.04g051200*, *Glyma.04g062400*, and *Glyma.04g073900*, which are involved in cell and seed development [12]. In various soybean crops, several CYP family members regulate seed weight, with significant increases in seed weight achieved by overexpressing *GmCYP78A5* (*Glyma.05g019200*) and *GmCYP78A72* (*Glyma.19g240800*) in soybean and *Arabidopsis* [13,14]. *GmCYP82C4* (*Glyma.04g035500*) is significantly associated with soybean seed weight [15]. *GmKIX8-1* (*Glyma.17g112800*), encoding a KIX domain-containing protein, has been verified as the causative gene for the major QTL (*qSw17-1*) of seed weight. The functional loss of *GmKIX8-1* results in larger seeds and leaves in soybean [16]. *GmGA20OX* (*Glyma.07g08950*), encoding gibberellin 20 oxidase 2, affects seed development by regulating gibberellin (GA) synthesis [17]. Overexpression of *GmGA20OX* is positively correlated with seed weight in transgenic *Arabidopsis* plants [18]. Moreover, *Arabidopsis* overexpressing the *protein phosphatase 2C-1* (*PP2C-1*) gene from wild soybean ZYD7 also shows significantly enhanced seed weight/size through larger cell size [19]. Additionally, a few major or stable QTLs across several environments have been reported, limiting their effective use in improving phenotypes through marker-assisted selection.

The QTLs controlling 100SW and seed size have been identified by genome-wide association studies (GWASs) in various crops [20,21,22]. Studies in soybean have primarily relied on chip-based single-nucleotide polymorphism (SNP) arrays to uncover significant associations between genetic markers and seed size [23,24,25,26]. Several notable studies have identified QTLs and candidate genes contributing to variations in 100SW, providing valuable insight into breeding strategies. However, these studies were often limited by the fixed and relatively sparse nature of SNP arrays, potentially missing rare or novel variants that could be critical for explaining additional phenotypic variance. In contrast, the advent of high-throughput sequencing technologies has revolutionized genetic discovery in plant genomics. Re-sequencing allows for the identification of millions of SNPs across the genome, offering much denser and more comprehensive coverage than chip-based methods. GWASs utilizing over 1 million SNP markers derived from whole-genome resequencing data in other crops have isolated new QTL regions and candidate genes for traits, such as grain size, plant height, and drought tolerance, demonstrating the power of high-density SNP markers in revealing genetic complexity [27,28]. Building on these advancements, our study aimed to apply a similar high-resolution approach to soybean 100SW. By imputing re-sequencing data from over 420 accessions in the Korean soybean core collection, we intend to leverage approximately 3.8 million high-density SNP markers for our GWAS analysis. This method has the potential to significantly enhance our understanding of the genetic landscape associated with seed size, moving beyond the limitations of previous studies to identify novel QTL regions and candidate genes that have remained elusive to date. First, it allows the detection of common and rare genetic variants that may contribute to variability in 100SW, thereby providing a more comprehensive genetic picture. Second, it enables the exploration of complex trait–gene interactions and epistatic effects that are often overlooked in less dense genetic maps. In summary, incorporating re-sequencing data and using high-density SNP markers in our GWAS analysis promises to expand our knowledge of the genetic determinants of soybean seed weight and establish a foundation for future genetic studies in soybean and other crops. This study successfully identified novel genes and loci associated with 100SW, including previously uncharacterized loci contributing to its natural variation. These findings provide new insight into the complex genetic architecture of 100SW and offer valuable targets for molecular breeding and genomic selection strategies aimed at improving soybean yield and quality.

## 2. Results

### 2.1. Analysis of Genetic Variation Between Phenotypes Related to Seed Yield

**Figure 1:**
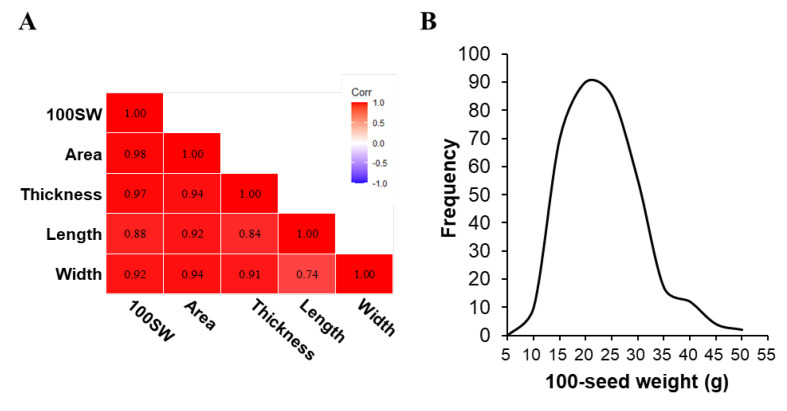
Phenotypic variation in 100-seed weight, and the correlations between the five seed agronomic traits in the Korean soybean core collection. (**A**) Pearson’s correlation coefficients estimated among five agronomic traits in the Korean soybean core collection. Numbers represent the value of the correlation coefficient between the row trait and the column trait. The red and purple boxes indicate positive and negative correlations between the two traits, respectively. 100SW: 100-seed weight; area: surface area parallel to the hilum; thickness: diameter vertical to the hilum; length: diameter parallel to the hilum; width: diameter from the hilum to the back of the seed. (**B**) Frequency distribution of soybean 100SW across all of the 344 accessions in 2016.

We investigated various seed phenotypic traits associated with soybean productivity, focusing on attributes such as area, width, length, thickness, and 100SW in 2017. As 100SW is a critical trait for improving soybean yield, we analyzed the correlation with seed morphological characteristics to determine their effect on productivity. The seed morphology of the Korean soybean core collection was phenotyped in four dimensions, including area, thickness, length, and width. Correlation analysis, conducted in 2017, revealed strong associations between 100SW and these morphological traits, with correlation coefficients of 0.98 for area, 0.97 for thickness, 0.88 for length, and 0.92 for width (Figure 1A). All traits showed consistently strong relationships with seed weight, indicating that seed size and weight are closely related and likely affected by similar genetic factors.

To evaluate the 100SW phenotypic variation in the soybean accessions across 2016, 2017, and 2021, we selected and analyzed phenotypic data from the Korean soybean core collection, including 344 accessions. The 100SW distributions were generally similar across the 3 years. The 100SW ranged from 7.0 to 48.3 g, with a mean ± standard deviation (SD) of 20.95 ± 7.24 g, in 2016; from 8.0 to 47.5 g, with a mean ± SD of 20.58 ± 7.39 g, in 2017; and from 6.4 to 41.9 g, with a mean ± SD of 18.79 ± 6.49 g, in 2021 (Figure 1B and Supplementary Figure S1). Skewness and kurtosis were < 1, indicating continuous variation and an almost normal distribution. This result highlights the wide variation in 100SW within this population, suggesting a large amount of genetic variation. The heritability (*h*^2^) of seed weight across the three years (2016, 2017, and 2021) was estimated to be 0.9863 (98.63%) (Supplementary Table S1), indicating that most of the phenotypic variation is attributable to genetic factors rather than environmental effects. This high heritability supports the stability of the identified loci across environments and suggests that genetic approaches are highly effective for improving or predicting 100SW in soybean.

### 2.2. Identification of Loci Associated with 100SW According to the GWAS

**Figure 2:**
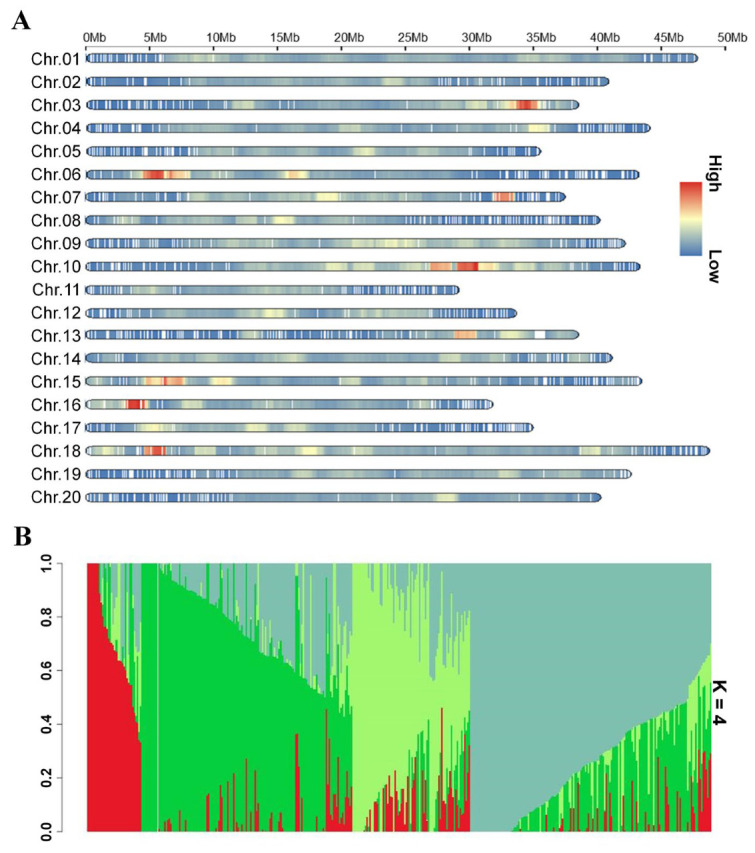
Distribution of high-quality SNPs across the soybean chromosomes. (**A**) SNP density plot chromosome-wise representing the number of SNPs within a 1 Mb window. The horizontal axis indicates chromosome length (Mb); the different colors depict SNP density. (**B**) Admixture analysis was conducted on a set of 422 soybean core collection accessions utilizing 4,406,436 SNP markers. Colors denote genetic ancestries (red, green, light green, and light blue).

The soybean core collection accessions were previously genotyped by whole-genome resequencing (SRA Accession: PRJNA555366) and are publicly available [29]. The 344 accessions from the Korean soybean core collection and 4.4 million high-density SNP markers were analyzed, and the SNP marker distributions are shown in Figure 2A. Averaging 223,641 SNPs per chromosome (Chr.), Chr. 18 had the maximum number of markers (407,804 SNPs), whereas Chr. 12 contained the minimum number (101,699 SNPs). Similarly, SNP density varied across Chr. 18, which had the highest SNP density (6996 SNPs/Mb), whereas Chr. 12 had the lowest SNP density (2448 SNPs/Mb). The higher density of SNPs on Chr. 18, with some being highly frequent, suggests that this chromosome has a higher gene density or has experienced more recombination events, leading to a greater accumulation of genetic variation. The lower number of SNPs on Chr. 12 suggests that this chromosome has relatively lower genetic diversity than the others. Resolving SNP density was sufficient to identify the 100SW-related loci in the Korean soybean core collection. The 344 accessions were grouped into four subpopulations (*K* = 4), as evidenced by the population structure obtained from ADMIXTURE, which was consistent with the findings from our previous study (Figure 2B) [30].

A preliminary GWAS was performed to validate the reliability of the analysis before proceeding with 100SW using the aforementioned two distinct sets of SNP markers, and known qualitative traits that induce white flower color in soybean. The genes *W1* and *Wm*, which control flower color in soybean, have been mapped to Chr. 13 [31,32,33]. The GWAS identified a significant peak on Chr. 13, which corresponded to the expected location of these genes, suggesting that the GWAS was stable and reliable (Supplementary Figure S2). GWAS was conducted to identify the genomic regions associated with soybean seed weight with data collected during the 3 years (2016, 2017, and 2021), enabling cross-environmental comparison of association signals. Manhattan and quantile-quantile (Q–Q) plots were generated to visualize genome-wide associations between SNPs and 100SW. Of the total 272 SNPs detected by a mixed linear model (MLM)-GWAS, 10 were consistently found to have significant associations (−log_10_(*p*) > 5) in the three environments (2016, 2017, and 2021) (Supplementary Figure S3). These SNPs, located on Chr. 3, 4, and 19, showed stable genetic effects on the seed weight trait, as they were repeatedly associated with the trait across different environmental conditions. Among them, eight SNPs were found on chromosome 3 and one each on Chr. 4 and 19. Although fewer SNPs were detected on chromosomes 4 and 19 than on chromosome 3, the signals from these loci appeared consistently across all three years. To further validate the allelic effects of the repeatedly identified SNPs, genotype-specific differences in 100SW were compared across the three environments. As shown in Supplementary Figure S4, the effect allele associated with changes in 100SW is indicated by underlining in the sequence alignment and the phenotypic separation among genotypes was consistent for each SNP, demonstrating that the effect allele exerted a similar influence in 2016, 2017, and 2021. The year-specific significance levels and phenotypic effect estimates for these SNPs are summarized in Table 1. Although the magnitude of the phenotypic effect varied slightly among years, the direction and relative strength of the allelic effects remained stable across environments, supporting the robustness of these loci. We next examined whether the eight SNPs on chromosome 3 represented independent loci by performing linkage disequilibrium (LD) analysis. The first two SNPs (snp.03.38576307 and snp.03.38576403) showed complete LD with each other (D′ = 1.0), indicating that they constitute a single QTL region. In contrast, the remaining six SNPs exhibited high LD among themselves (D′ > 0.97) but not with the first pair, forming a second, distinct LD block (Supplementary Figure S5). These results demonstrate that the eight SNPs on chromosome 3 cluster into two independent QTL regions rather than multiple unrelated loci. The genomic positions of the significant SNPs were compared with previously reported seed-weight QTLs in SoyBase Genome Browsers (https://soybase.org) (Supplementary Table S2). Interestingly, the eight SNPs on chromosome 3 also displayed two distinct patterns of effect size, further supporting the presence of two separate QTL regions on this chromosome. Notably, none of the identified SNPs overlapped with known seed-weight QTLs, suggesting that these loci may represent previously unreported genetic regions contributing to variation in 100SW.

### 2.3. Haplotypes for the Seed Weight Trait

**Figure 3:**
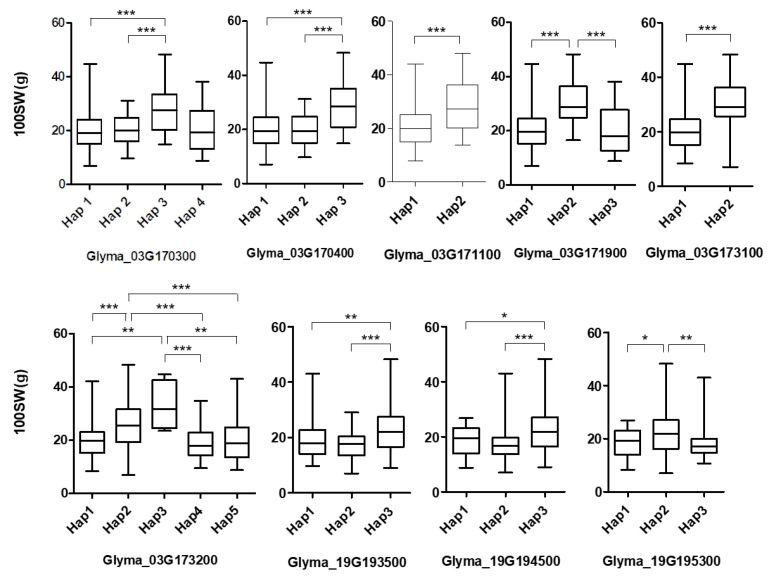
Haplotype analysis of the soybean seed weight trait. Boxplots display the 100SW values corresponding to the groups of different haplotypes in the nine evaluated locations. Hap1 represents the reference allele, while Hap2, Hap3, and Hap4 represent alternative alleles. Definition of statistical significance: *p* < 0.05 (*), <0.01 (**), <0.001 (***).

To search for genomic regions surrounding the SNPs, linkage disequilibrium was estimated, and potential candidate genes were mined within 100 kb upstream and downstream of the 10 significant SNPs that were detected across three years. Seventy-one potential candidate genes were identified in Arabidopsis based on the SoyBase database and the orthologues (Supplementary Table S3). The majority of these genes exhibited enrichment in the nuclear component within the cellular component category, alongside DNA binding in biological processes and nucleosome assembly in the molecular function categories. We searched for mutations, such as substitutions and indels, in the candidate gene regions of interest and revealed non-synonymous substitutions in 37 of the 71 candidate genes. GO enrichment analysis showed that the GWAS-predicted candidate genes were enriched in biological process, cellular component, and molecular function categories (Supplementary Figure S6), suggesting their potential involvement in diverse functional pathways related to the trait. Haplotype analysis was performed across the target regions to assess the variation associated with seed weight, revealing significant differences (*p* < 0.05) in 29 candidate genes (Supplementary Tables S3 and S4). To further refine the candidates, we conducted a functional annotation search to identify genes with potential roles in seed productivity. Based on previous studies and available functional data, we selected genes that have been suggested to be associated with seed yield or related agronomic traits. As a result, we narrowed down the list to nine genes that are most likely to contribute to seed productivity, including seed weight: *Glyma.03g170300*, *Glyma.03g170400*, *Glyma.03g171100*, *Glyma.03g171900*, *Glyma.03g173100*, and *Glyma.03g173200* on Chr. 3, and *Glyma.19g193500*, *Glyma.19g194500*, and *Glyma.19g195300* on Chr. 19 (Supplementary Table S3). Four haplotype alleles were identified in *Glyma.03g170300,* where accessions with Hap3 (TCACA, alternative allele) revealed higher mean 100SW than those with Hap1 (AAACG, reference allele) in the soybean population (Figure 3 and Supplementary Table S4). Similarly, the Hap3 alleles (CGTCCGTAAT, alternative) in *Glyma.3g170400*, the Hap2 alleles (G, alternative) in *Glyma.03g171100*, the Hap2 alleles (GG, alternative) in *Glyma.03g171900*, the Hap2 alleles (A, alternative) in *Glyma.03g173100*, and the Hap3 (TCACTACTAA, alternative) alleles in *Glyma.03g173200* were associated with higher 100SW compared to their respective reference alleles. The Hap3 alleles (AA, alternative) in *Glyma.19g193500* and *Glyma.19g194500* (TC, alternative), and the Hap2 alleles (GTC, alternative) in *Glyma.19g195300*, were associated with higher 100SW on Chr. 19 compared to their respective reference alleles. These findings suggest potential roles of these genes in regulating key aspects of seed productivity, providing valuable insight into the genetic mechanisms underlying these agronomic traits.

### 2.4. Candidate Gene Prediction and Further Analyses

**Figure 4:**
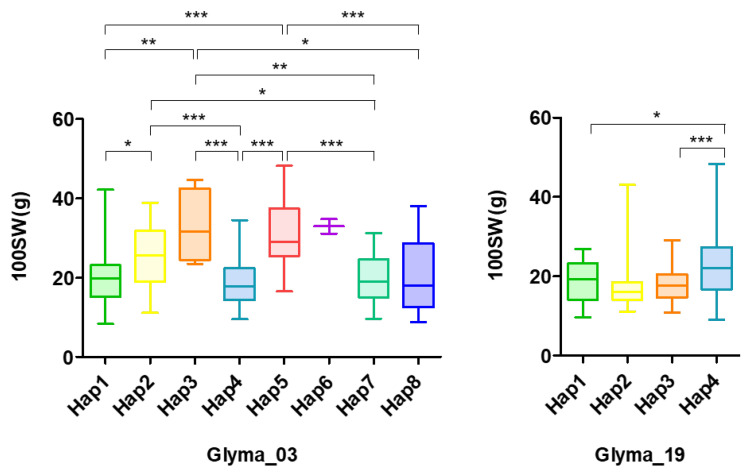
Allelic combinations on Chr. 3 and Chr. 19 enhance the Korean core soybean collection 100SW. Effect of alleles at the six and three genes of chromosomes 03 and 19, respectively. *Glyma_03G170300*, *Glyma_03G170400*, *Glyma_03G171100*, *Glyma_03G171900*, *Glyma_03G1731000*, and *Glyma_03G173200* for Chr. 03, and *Glyma_19G193500, Glyma_19G194500*, and *Glyma_19G195300* for Chr. 19. See Supplementary Table S5. Significance at *p* < 0.05 (*), <0.01 (**), <0.001 (***), respectively.

Identification and utilization of candidate genes are important objectives of GWAS to improve soybeans. In the six genes of Chr. 3 and three genes of Chr. 19, the presence of a specific nucleotide variant consistently indicated a noticeable increase in 100SW (Figure 4). An allelic stacking analysis was conducted to assess the simultaneous effects of all six or seven variants. As expected, the combined haplotype analysis revealed an overall increase in 100SW, similar to the results observed from the individual gene analyses (Figure 4 and Supplementary Table S5). Interestingly, the haplotype containing the variant in the *Glyma.03g173200* gene exhibited a higher 100SW than the haplotype where variants were present across all six genes. An allelic stacking analysis with the Chr. 19 genes showed that the highest 100SW was observed in the haplotype where variants were simultaneously present in all three genes. This result suggests that these genes potentially modulate soybean seed weight through expression-level and allelic effects.

## 3. Discussion

Soybean seed weight is a key factor in determining yield and a typical quantitative trait controlled by multiple genes with small effects that are highly influenced by environmental factors, leading to significant variations in heritability [4,5]. Small population sizes and low-density markers in soybean studies have resulted in many loci having minor effects on seed weight, often mapped to relatively large intervals. As most of the reported QTL/QTNs are environmentally specific or located at large genomic intervals, breeders prefer to use QTL/QTNs with small genomic intervals in molecular breeding programs [34,35,36]. Therefore, it is important to identify the environmentally stable seed weight QTL/QTNs within a small region for marker-assisted selection. In the present study, the Korean soybean core collection of 344 accessions was used to map and validate key loci associated with seed weight, utilizing 4.4 million high-density data over 3 years. The phenotypic evaluations for 100-seed weight were conducted in year 2016, 2017 and 2021 under distinct environmental conditions, including differences in cultivation sites and seasonal climate patterns. Notably, the 2021 growing season experienced disease pressure that was not observed in the earlier seasons. Because these environmental and pathological factors had direct impacts on phenotypic expression, combining the datasets in a multi-environment joint GWAS would have confounded environment-specific effects rather than improving detection power. Therefore, we conducted GWAS independently for each year and focused on QTLs that were consistently detected across years, enabling the identification of stable loci with robust genetic influence on 100SW. In the GWAS conducted using a MLM, several loci for 100SW were distributed on Chr. 3, 4, and 19 across the different environments, suggesting that these loci have a stable genetic effect on soybean seed weight (Table 1 and Supplementary Figure S3). When the positions of the detected loci were compared with the QTLs in the Soybase database, the loci identified in this study were newly reported in relation to soybean seed weight.

The genes controlling seed weight in soybeans are still largely unknown, with a limited number of related genes reported [13,16,19,37]. A *PP2C* gene (*Glyma.02G278100*) was identified within locus *qSS2* as being related to soybean seed weight/size, and a homologous gene (*Glyma.17g221100*) regulates seed weight in soybean [19]. Several CYP family members have been revealed to be involved in the seed weights of soybeans and other crops [13,14,15]. *GmKIX8-1* (*Glyma.17G112800*) has been validated within a major QTL (*qSw17-1*) of seed weight, and its loss of function leads to larger seed and leaf size in soybean [16]. *GmGA20OX* (*Glyma07g08950*), encoding gibberellin 20 oxidase 2, affects seed development by regulating GA synthesis [17]. Our research mined several key loci and potential candidate genes, but none of the candidates has been reported to in/directly control seed weight in soybeans. The Korean core collection accession with the alternative allele of genes in Chr. 3 exhibited higher soybean 100SW than the accessions with a reference allele. One of the candidates in this study (Figure 3 and Supplementary Table S3), *Glyma.03g170300* homologous to *At2g20180*, is a *phytochrome-interacting factor 1* (*PIF1*). The PIF proteins are crucial in regulating growth and development by participating in various signaling pathways, including light, hormones, temperature, and abiotic and biotic stressors, enabling plants to adapt to environmental changes [38]. PIF1, a basic helix–loop–helix transcription factor in *Arabidopsis*, is responsible for activating a set of genes whose products inhibit the completion of seed germination and regulate genes involved in abscisic acid and GA biosynthesis, thereby affecting the production and metabolism of their bioactive forms [39,40]. The *Glyma.03g170300* gene likely influences seed weight by regulating hormonal pathways critical in seed development. *Glyma.03g171100*, annotated by heat shock cognate 70 kDa protein 2, played a role in seed production in this study. In a previous study, pod size of soybean (*GmPSS8*), a member of the heat shock protein 70 (HSP70) family, was identified as a candidate gene influencing pod length within a major QTL for this trait [41]. GmPSS8 regulates pod length in soybeans by controlling cell proliferation through transcriptional regulation during the early stages of pod development. As the pod is a critical organ for producing seeds, understanding the mechanisms of pod development provides valuable insight and potential strategies for enhancing yield potential in soybeans. In this study, the *Glyma.03g171900* gene was identified as a candidate, functionally annotating a short-chain dehydrogenase. The short-chain dehydrogenases/reductases (SDRs) are enzymes of great functional diversity, and in silico analysis of SDR proteins and gene expression profiling indicated high expression of many *SDR* genes in floral tissues and during seed germination stages, highlighting their potential roles in reproduction and seed development [42,43]. *Glyma.03g173100* and *Glyma.03g173200* are homologous to *At2g20180* and annotated as C2H2 and C2HC zinc finger superfamily proteins. Zinc finger proteins have been classified into different categories based on the number and order of the cysteine (Cys) and histidine [33] residues that bind the zinc ion; Cys2/His2 (C2H2), C2C2, C2HC, C2C2C2C2, C2HCC2C2, and the CCCH type [44]. Among the proteins, the C2H2 zinc finger proteins constitute one of the largest families of transcriptional regulators in plants and are the most extensively studied [45,46]. The zinc finger protein ZFP207 (C2H2 type) suppresses GA synthesis by binding to the OsGA20ox2 promoter, resulting in dwarfism, short grains, and panicle phenotypes in rice [47]. Zinc finger proteins affect seed weight, size, and yield in plants through a variety of mechanisms [48]. For example, *GmPLATZ*, a zinc-finger transcriptional regulator, increases seed size and weight in soybeans by directly binding to promoters and activating cyclin genes and *GmGA20OX*, which are positively correlated with seed weight [18,48]. This study showed that the orthologues of *GmPLATZ* in rice and *Arabidopsis* perform similar functions in seeds, suggesting the novel involvement of *GmPLATZ-GmGA20OX*/cyclins in regulating seed size and weight, and serving as promising targets for crop breeding to enhance agronomic traits. Based on these results, the genes identified on Chr. 3 are likely to have a positive influence on soybean 100SW.

Similarly, the aforementioned genes on Chr. 19 are likely to have significant implications for 100SW based on their functional annotation. In plants, basic-leucine zipper (bZIP) transcription factors function in many biological processes, including growth, flower maturation, seed development, and stress in soybeans [49]. *GmbZIP97* and *GmbZIP159* are involved in GA biosynthesis, which is associated with soybean 100SW and seed number [50]. CRISPR/Cas9-generated *gmga3ox1* knockout mutants, which encode a key GA biosynthetic enzyme, decrease the content of bioactive GAs in soybean leaves and lower seed weight, elucidating the important role of *GmGA3ox1* in soybean yield, thereby promoting seed yield by upregulating *GmbZIP97* and *GmbZIP159*. In addition, the GmbZIP transcription factors abscisic acid-responsive element binding protein (AREB)3-1 (GmbZIP35), AREB3-2 (GmbZIP57), and GmbZIP92 regulate specific seed development processes in coordination with leafy cotyledon1, a key transcription factor governing embryo morphogenesis, and seed maturation [51]. In light of these findings, *Glyma.19g194500*, functionally annotated as bZIP transcription factor 4, is hypothesized to influence seed development and 100SW. Similar to *GmbZIP97* and *GmbZIP159*, *Glyma.19g194500* may contribute to GA-mediated regulatory networks, which are critical for determining seed weight and yield in soybeans. On the other hand, *Glyma.19g195300* is annotated as a kinesin-like protein in soybean. The *short grain length* (*SGL*) gene encodes a kinesin-like protein with transcriptional activation activity in rice, and an *SGL* mutation affects GA metabolism-related gene expression, resulting in dwarfism and reduced glume cell length [52]. This observation suggests that *SGL* regulates grain length and plant height in rice by influencing GA synthesis and response pathways. From these findings, it was anticipated that *Glyma.19g194500* and *Glyma.19g195300* would be involved in seed development and contribute to seed yield, potentially through their effect on GA pathways. Thus, it can be inferred that the genes identified in Chr. 3 and 19 are likely to influence seed development and production through their involvement in GA biosynthesis and signaling pathways, as supported by their functional annotations and known roles in GA-mediated regulatory networks. These findings suggest their potential as key targets for improving soybean seed traits. Furthermore, allelic stacking analysis for each chromosome revealed that the combined presence of favorable alleles significantly enhanced 100SW, reinforcing the hypothesis that these genes contribute to seed productivity through additive genetic effects. Specifically, the haplotype containing the *Glyma.03g173200* variant on Chr. 3 exhibited the highest 100SW among single-gene haplotypes, while the presence of favorable alleles across all three genes on Chr. 19 resulted in the maximum 100SW, suggesting a cumulative effect of these variants on seed weight. In this study, we identified the stable loci on Chr. 3 and 19 utilizing the 100SW data from the Korean soybean core collection, along with high-density SNP markers and GWAS assays. These findings overcame the limitations of previous studies that used lower-density markers. 

Although haplotype analysis was primarily used to identify candidate genes, we also performed RNA-seq analysis comparing large-seed and small-seed accessions. However, expression patterns were inconsistent among cultivars, and no statistically significant differential expression was observed for several genes. Therefore, these data were not included in the present study. Nevertheless, these preliminary results provide a valuable basis for further validation. Future research will focus on verifying the expression of these candidate genes through quantitative PCR and functional characterization via gene knockout or overexpression approaches. In addition, future analyses may incorporate multi-locus GWAS approaches to refine and validate the association signals detected in this study. Integrating these candidate genes into breeding programs will enhance soybean yield and adaptability under diverse conditions, demonstrating the utility of high-density genomic data for efficient soybean improvement.

## 4. Materials and Methods

### 4.1. Plant Materials and Phenotypic Evaluation

The Korean soybean core collection, comprising 422 accessions, was cultivated at the National Institute of Crop Science (NICS) in 2016, 2017, and 2021 [29]. We investigated the phenotypes of 344 accessions, excluding accessions with insufficient seed volume. Mature seeds for each accession were harvested and naturally dried to a stable weight at room temperature. The 100 seeds selected at random from each of the accessions were measured using an electronic balance across the 3 years, and their weights were recorded as 100SW (g). The seed phenotypes were measured at the National Institute of Agricultural Sciences in 2017 and described in a previous study [53] (Figure 1A). In brief, 100 seeds were arranged within a 210 × 297 mm area, with one top view and four side images captured. ImageJ was used to measure traits, such as area, width, height, and thickness, and coordinate values were used to align the top and side images. Flower color was observed in the experimental fields of the NICS, measured during the R2–R3 growth stages.

### 4.2. Genome-Wide Association Study and Haplotype Analysis

The GWAS and haplotype analysis for 100SW were conducted using the whole-genome resequencing data and the publicly available SRA Accession number PRJNA555366, as described previously [29]. From a total of 4,472,823 high-density SNPs, monomorphic sites, low-coverage variants, and SNPs with a minor allele frequency < 0.05 were removed [54]. In addition, SNPs with >5% missing data across accessions were eliminated to reduce the effects of incomplete genetic information; therefore, 3,897,471 SNPs were used for the GWAS. Hardy–Weinberg equilibrium *p*-values < 1 × 10^−100^ were considered, and Mendelian errors identified using recorded pedigree relationships were addressed by setting the genotypes of individuals with erroneous SNPs to “missing”. To minimize false-positive results and increase statistical power, GWAS analyses were performed using QTLmaxV4 [55] with a MLM and default settings, accounting for both population structure and kinship relationships. The MLM was represented by the equation y = Xβ + Zu + ε, where y denotes the observed phenotype, “β” and “u” represent fixed effects (marker information) and random effects (individual information), respectively, ε is the random residual effect, and X and Z are the associated design matrices. The random effects model assumes that “u” follows a normal distribution with a mean of zero and variance–covariance matrix G (u ~ N(0, G)), and ε follows a normal distribution with a mean of zero and variance. Significant markers were selected from the GWAS results based on a threshold of −log10(*p*) > 5, and each gene within the range of these markers was identified based on the Glycine max reference genome Wm82.a2.v1 available in SoyBase Genome browsers (https://soybase.org). The inclusion of significant markers within the coding sequence of candidate genes was verified.

Linkage disequilibrium (LD) analysis was performed using LDBlockShow with default parameters to visualize LD patterns and identify QTL-level clustering among the significant SNPs on chromosome 3. Pairwise LD values were calculated based on the filtered SNP dataset.

### 4.3. Statistical Analysis

Analysis of variance (ANOVA) of seed size- and shape-related traits was conducted with Origin2018 software (Origin Laboratories, Northampton, MA, USA). The descriptive statistics and correlation coefficients were analyzed using Microsoft Excel (Microsoft Corp., Redmond, WA, USA) and JAMOVI software (version 2.6.44) (https://www.jamovi.org). Broad sense heritability was calculated via the lme4 package (version 1.1-25) in R language (version 4.4.2; R Foundation for Statistical Computing, Vienna, Austria). The ANOVA or *t*-test of the phenotypic variance among the haplotype groups was conducted using GraphPad Prism 10.3.1 (GraphPad Inc., La Jolla, CA, USA).

## Figures and Tables

**Table 1:** Year-specific significance levels and phenotypic effect estimates of detected SNPs.

Marker ^a^	Chr. ^b^	Year	log10(*p*) ^c^	Effect Allele ^d^ (Ref->Alt)	Phenotypic Effect ^e^ (Mean Difference, g)
snp.03.38576307	3	2016	6.45	A->T	8.3
		2017	6.71		8.56
		2021	5.05		7.22
snp.03.38576403	3	2016	6.45	A->G	8.3
		2017	6.71		8.56
		2021	5.05		7.22
snp.03.38685095	3	2016	5.97	C->T	4.69
		2017	5.57		4.4
		2021	5.14		4.33
snp.03.38685383	3	2016	5.97	T->A	4.69
		2017	5.57		4.4
		2021	5.14		4.33
snp.03.38685486	3	2016	5.97	A->G	4.69
		2017	5.57		4.4
		2021	5.14		4.33
snp.03.38685655	3	2016	5.97	T->C	4.69
		2017	5.61		4.4
		2021	5.08		4.33
snp.03.38686042	3	2016	5.97	T->G	4.69
		2017	5.57		4.4
		2021	5.14		4.33
snp.03.38686093	3	2016	5.97	T->C	4.69
		2017	5.57		4.4
		2021	5.14		4.33
snp.04.40965491	4	2016	7	G->A	6.86
		2017	6.25		6.65
		2021	5.62		6.21
snp.19.45174797	19	2016	5.38	G->A	9.33
		2017	5.54		9.5
		2021	6.8		8.52

^a^ Marker: SNP markers repeatedly identified across three years; ^b^ Chr.: chromosome; ^c^ log10(*p*): year-specific significance levels (−log_10_(*p*)) of GWAS results (Supplementary Figure S3); ^d^ Effect allele (Ref→Alt): lists of the reference and alternative nucleotides, and underlined nucleotide indicating the effect allele responsible for the change in 100SW; ^e^ Phenotypic effect: mean difference in 100SW between Ref and Alt genotypes indicating the direction and magnitude of allelic effects.

## Data Availability

The raw sequencing data from this study have been deposited at The National Center for Biotechnology Information (https://www.ncbi.nlm.nih.gov) under Accession number: PRJNA555366.

## Supplementary Materials

The following supporting information can be downloaded at: https://www.mdpi.com/article/10.3390/ijms262411921/s1.
