# Supplementary material for: Identification of Novel Genetic Loci Related to 100-Seed Weight in the Korean Soybean Core Collection Using a Genome-Wide Association Study
Source: Int J Mol Sci. 2025 Dec 10;26(24):11921. doi: 10.3390/ijms262411921 (PMC12732426; doi:10.3390/ijms262411921)
Supplement: Supplementary file 1 [file ijms-26-11921-s001.zip › Supplementary Figures.pdf]

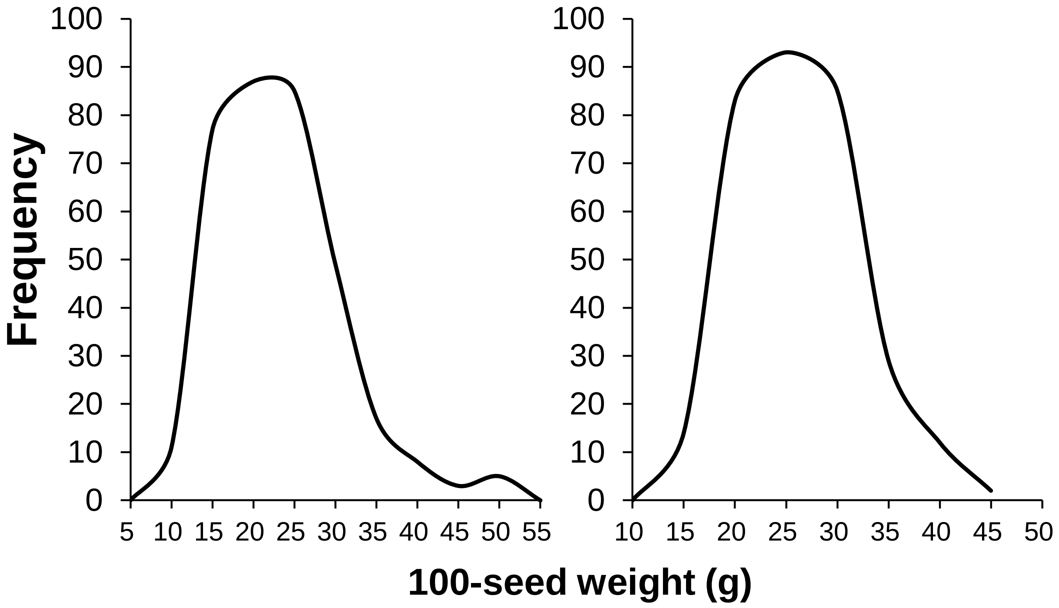

Supplementary Figure S1. Phenotype variation in 100-seed weight of Korean soybean core collection. Frequency distribution of soybean 100-seed weight (100SW) across all of the 344 accessions in the year 2017 (left) and 2021 (right). The x-axis represents 100-seed weight grouped in 5-g intervals; 5 indicates 0 ≤ 100SW < 5, 10 indicates 5 ≤ 100SW < 10, and so on.

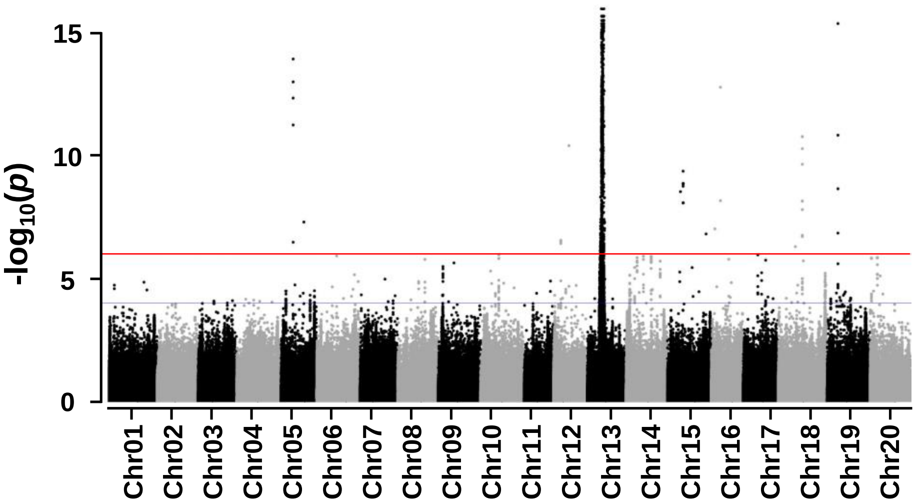

Supplementary Figure S2. Manhattan plot of genome-wide association study (GWAS) analysis between SNPs and flower color. The analysis was conducted using 4.4 million high-density SNP markers. The y-axis represents the level of statistical association (log10(*p*)) for each SNP, arranged by chromosomal location along x-axis. The red horizontal line indicates the significance threshold (-log10(*p*) > 6) used in this study.

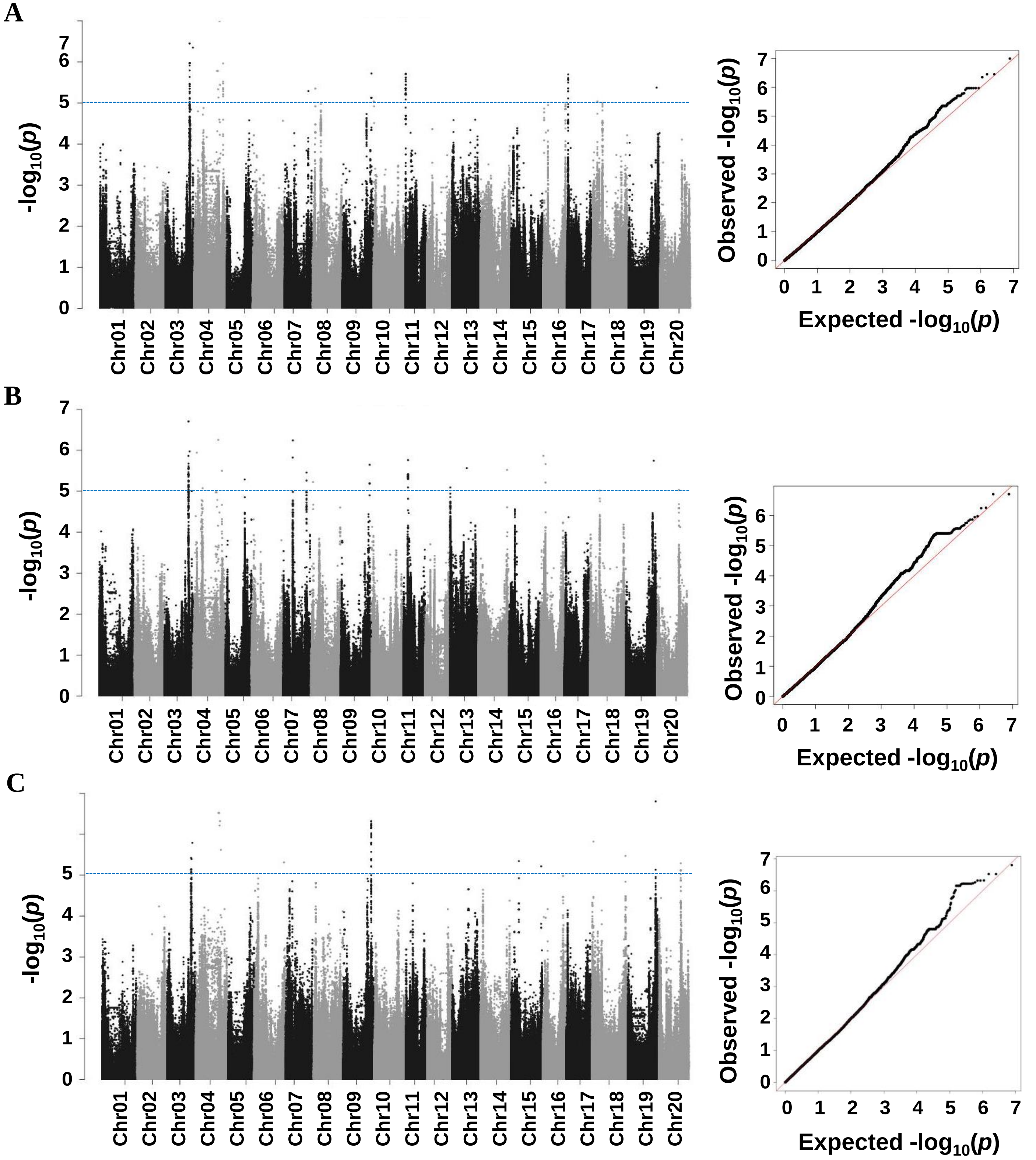

Supplementary Figure S3. GWAS for 100SW in Korean soybean core collection. Manhattan plots and quantile-quantile (Q-Q) plots of GWAS for 100SW in Korea core collection from year 2016 (A), 2017 (B) and 2021 (C) filed trials. GWAS results are presented by negative log10 P values against position on each 20 chromosomes. The blue dotted line indicates the suggestive significance threshold (-log10(*p*) > 5). The summary information of the SNPs significantly associated with 100SW in the GWAS. Quantile-quantile plot; the x-axis and y-axis indicate the negative log-scale of the expected *p* values for each SNP and the negative log-scale of the observed *P* values, respectively. A straight line indicates the expected results under Hardy-Weinberg equilibrium. (C) Chr; chromosome.

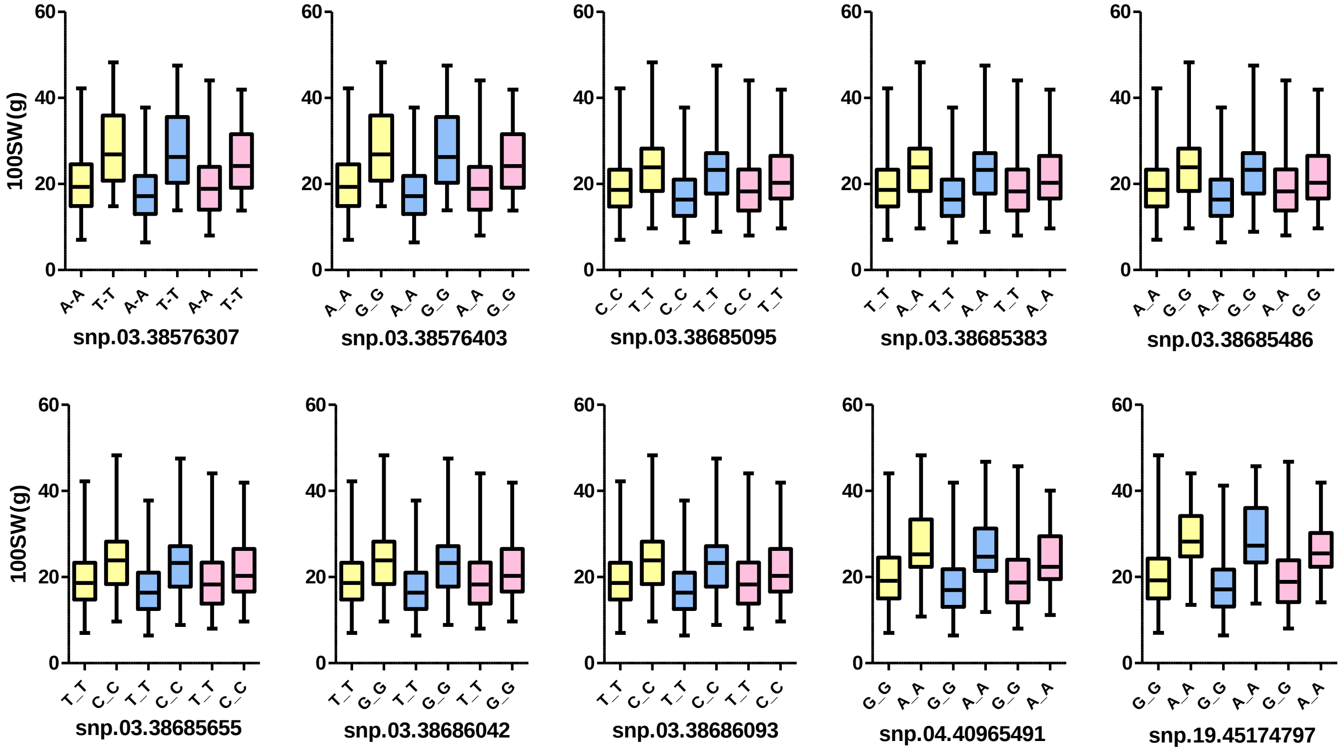

Supplementary Figure S4. Mean 100SW of SNP genotypes across three years. Bar plots showing the mean 100-seed weight of each SNP genotype in 2016 (yellow), 2017 (blue), and 2021 (pink).Each panel corresponds to one of the repeatedly detected SNPs listed in Table 1. All Ref vs. Alt genotype comparisons within each year were statistically significant according to a *t*-test ($p < 0.0001$).

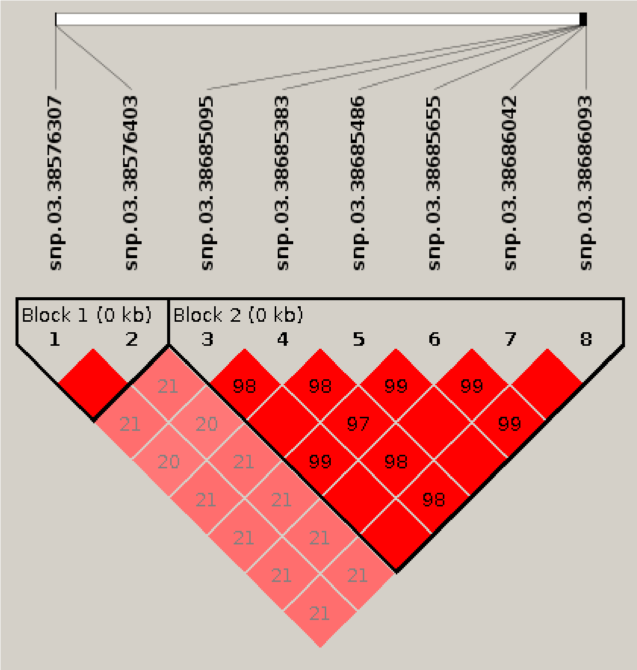

Supplementary Figure S5. LD structure of the eight SNPs on chromosome 3.Pairwise D′ values are shown for the eight repeatedly detected SNPs. Two LD blocks were identified: Block 1 (SNPs 1–2) and Block 2 (SNPs 3–8), indicating the presence of two independent QTL regions for 100-seed weight.

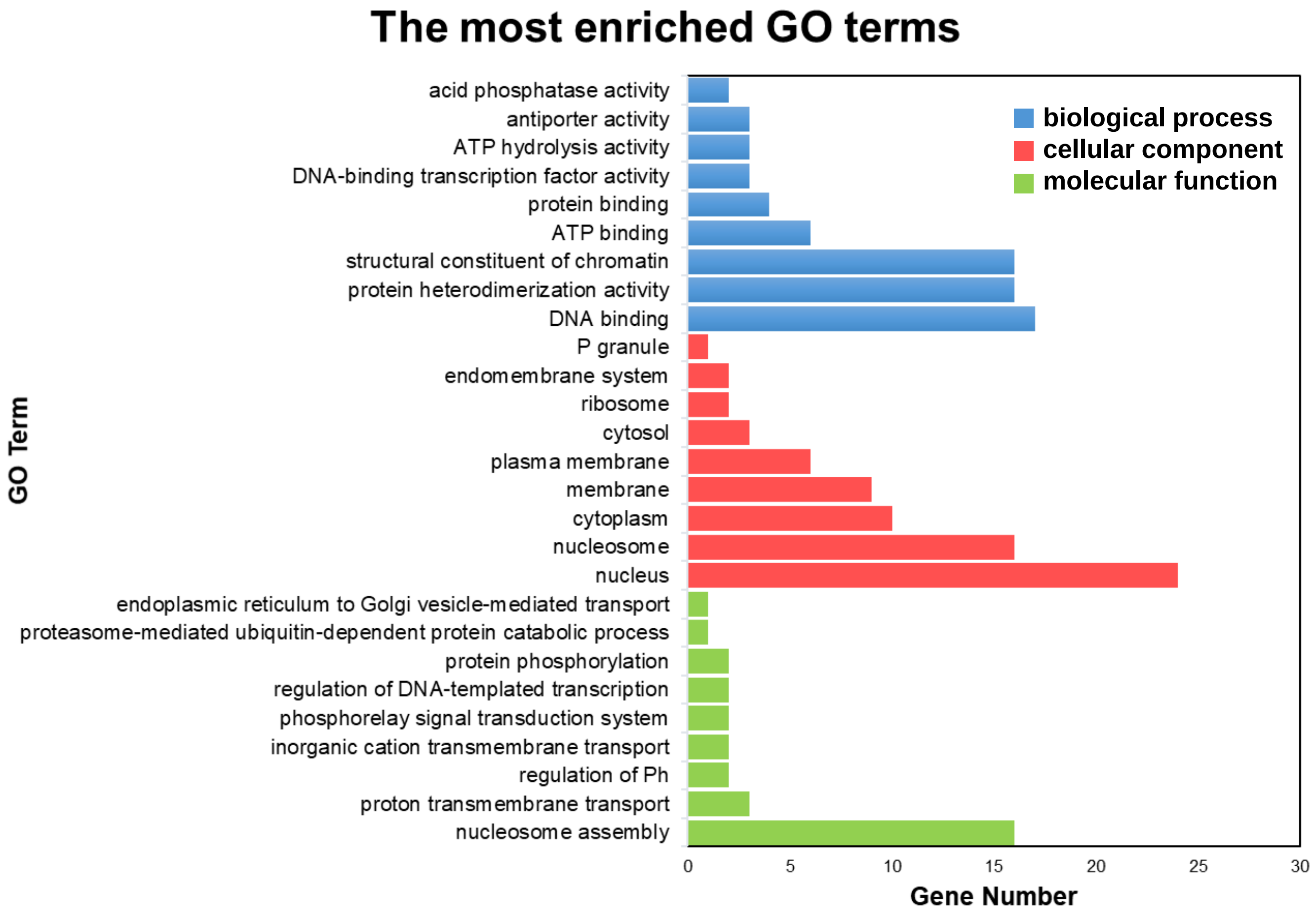

Supplementary Figure S6. Gene ontology (GO) enrichment analysis of the 71 potential candidate genes predicted by GWAS. Three categories of biological process, cellular component, and molecular function for the differentially expressed genes.
